# Room-Temperature Photoluminescence Mediated by Sulfur Vacancies in 2D Molybdenum Disulfide

**DOI:** 10.1021/acsnano.3c02103

**Published:** 2023-07-07

**Authors:** Yiru Zhu, Juhwan Lim, Zhepeng Zhang, Yan Wang, Soumya Sarkar, Hugh Ramsden, Yang Li, Han Yan, Dibya Phuyal, Nicolas Gauriot, Akshay Rao, Robert L. Z. Hoye, Goki Eda, Manish Chhowalla

**Affiliations:** † Department of Materials Science & Metallurgy, University of Cambridge, 27 Charles Babbage Road, Cambridge CB3 0FS, United Kingdom; ‡ Cavendish Laboratory, University of Cambridge, Cambridge CB2 1TN, United Kingdom; § Department of Physics, National University of Singapore, 2 Science Drive 3, 117551, Singapore; ∥ Division of Material and Nano Physics, Department of Applied Physics, KTH Royal Institute of Technology, Stockholm, SE-106 91, Sweden; ⊥ Inorganic Chemistry Laboratory, Department of Chemistry, University of Oxford, South Parks Road, Oxford OX1 3QR, United Kingdom; # Department of Chemistry, National University of Singapore, 3 Science Drive 3, 117543, Singapore; ∇ Centre for Advanced 2D Materials, National University of Singapore, 6 Science Drive 2, 117542, Singapore

**Keywords:** monolayer molybdenum disulfide, sulfur vacancy generation, room-temperature defect-mediated emission, long-lived localized exciton, sulfur vacancy passivation

## Abstract

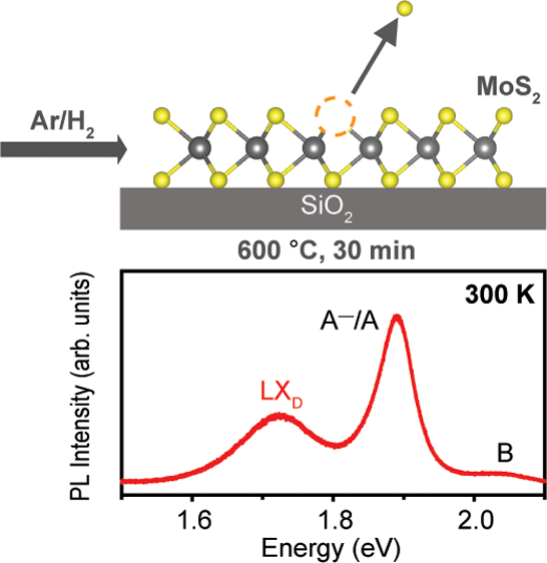

Atomic defects in monolayer transition metal dichalcogenides (TMDs) such as chalcogen vacancies significantly affect their properties. In this work, we provide a reproducible and facile strategy to rationally induce chalcogen vacancies in monolayer MoS_2_ by annealing at 600 °C in an argon/hydrogen (95%/5%) atmosphere. Synchrotron X-ray photoelectron spectroscopy shows that a Mo 3d_5/2_ core peak at 230.1 eV emerges in the annealed MoS_2_ associated with nonstoichiometric MoS_*x*_ (0 < *x* < 2), and Raman spectroscopy shows an enhancement of the ∼380 cm^–1^ peak that is attributed to sulfur vacancies. At sulfur vacancy densities of ∼1.8 × 10^14^ cm^–2^, we observe a defect peak at ∼1.72 eV (referred to as LX_D_) at room temperature in the photoluminescence (PL) spectrum. The LX_D_ peak is attributed to excitons trapped at defect-induced in-gap states and is typically observed only at low temperatures (≤77 K). Time-resolved PL measurements reveal that the lifetime of defect-mediated LX_D_ emission is longer than that of band edge excitons, both at room and low temperatures (∼2.44 ns at 8 K). The LX_D_ peak can be suppressed by annealing the defective MoS_2_ in sulfur vapor, which indicates that it is possible to passivate the vacancies. Our results provide insights into how excitonic and defect-mediated PL emissions in MoS_2_ are influenced by sulfur vacancies at room and low temperatures.

Monolayer or two-dimensional transition metal dichalcogenide (2D TMD) semiconductors have strong light–matter interactions.^1,2^ This results in high absorption coefficients,^3^ photoluminescence (PL) from tightly bound excitons,^4^ and valley-selective circular dichroism due to their noncentrosymmetric crystal structure.^5^ Further, van der Waals interlayer interactions allow the creation of vertical heterostructures with tunable optoelectronic phenomena.^6^ As a result, 2D TMDs are interesting for electronic devices, photovoltaics, and nanophotonics.^7^ However, monolayer TMDs prepared by mechanical exfoliation or chemical vapor deposition (CVD) possess a native defect density of ∼10^13^ cm^–2^.^8,9^ These defects are mostly chalcogen vacancies that cause changes in electronic structure by forming in-gap states and carrier doping.^10,11^ Monolayer TMDs are surprisingly tolerant of chalcogen vacancy defects. That is, they can host up to 1 × 10^15^ cm^–2^ chalcogen vacancies without significant distortion to their atomic structure.^12^ Above this concentration, however, significant damage to the structure occurs, and disordered regions start to appear. Various approaches to generate defects, such as *in vacuo* annealing,^13^ electron beam irradiation,^14^ focused X-ray beam irradiation,^15,16^ and focused ion beam exposure,^17,18^ have been reported. Controlled defect creation in 2D TMDs can induce single photon emission,^19,20^ enhance electro-/photocatalysis,^21,22^ and enable sensing.^23^ However, defects reduce the performance of electronic devices^24^ and therefore require minimization or effective passivation.^25,26^

One way to investigate changes in the electronic structure of 2D TMD semiconductors due to vacancy defects is to characterize the absorption and PL from defect-induced in-gap states. Absorption from such defect states has been observed at room temperature (RT ∼300 K),^27^ whereas light emission is typically observed at low temperatures at which excitons are bound to defect states to facilitate radiative recombination.^28,29^ As a result of this strong localization, the defect-mediated emission generally exhibits long lifetimes.^30^ In this work, we find that it is possible to observe emission from trapped excitons at RT when defect density (*n*_d_) is in the range of 10^13^ cm^–2^ < *n*_d_ < 10^15^ cm^–2^. We explored the influence of temperature and excitation power on localized emission. Time-resolved PL measurements were also been conducted. Further, we show that sulfur vacancies can be passivated with exposure to sulfur vapor and compare optical properties of defective and passivated samples to gain insight into how concentration of defects influences the optoelectronic properties of monolayer MoS_2_.

## Results and Discussion

**Figure 1:**
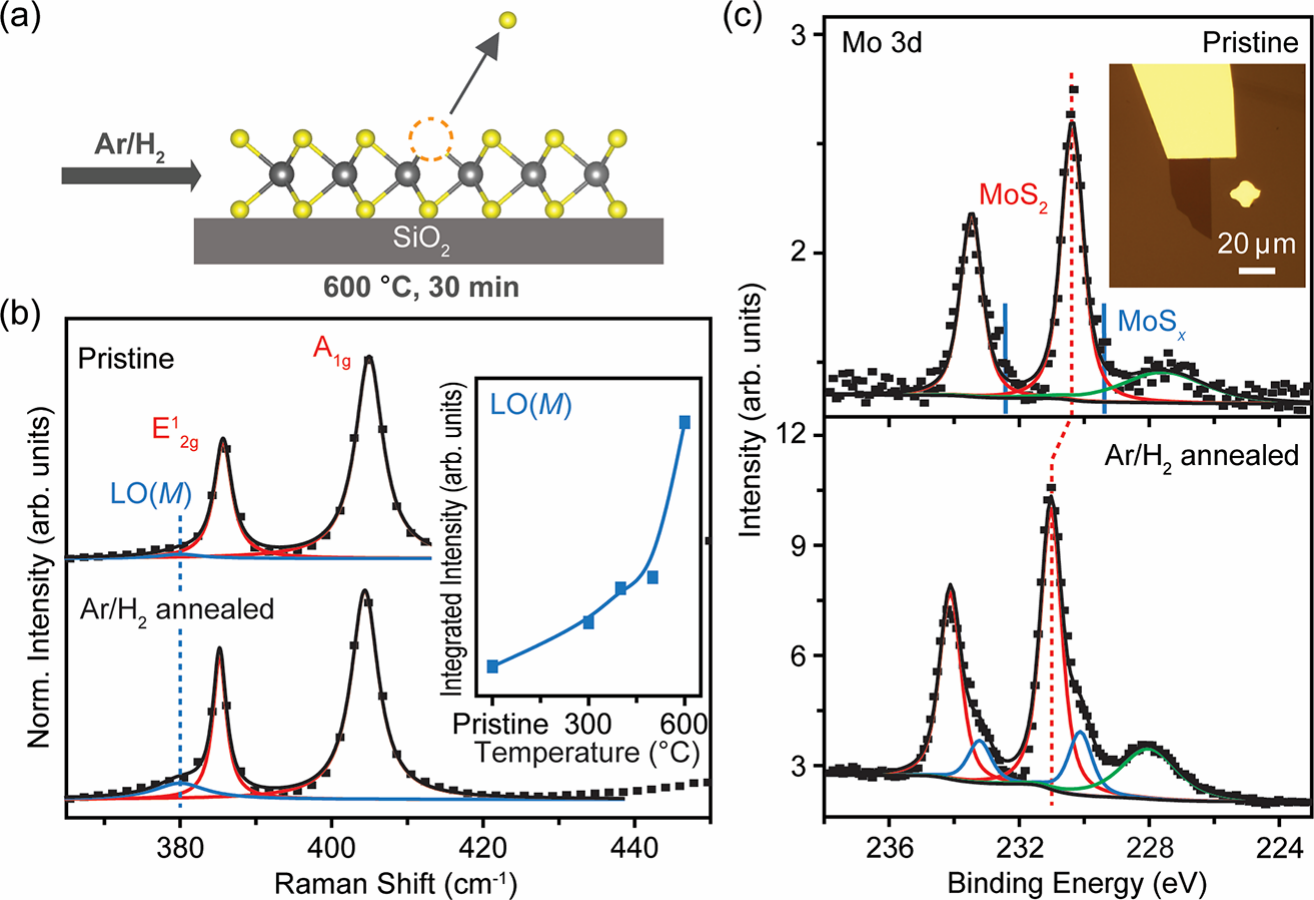
Defect generation in monolayer MoS_2_ by annealing in an Ar/H_2_ (95%/5%) atmosphere. (a) Schematic of sulfur vacancy generation through Ar/H_2_ annealing. (b) Raman spectra of pristine and 600 °C Ar/H_2_ annealed MoS_2_, normalized to the intensity of the Si reference peak. Pristine MoS_2_ shows an E^1^_2g_ in-plane mode and an A_1g_ out-of-plane mode. The low-frequency shoulder on the left of the E^1^_2g_ mode indicates the LO(*M*) defect mode, denoted in blue. The increased intensity of the defect mode in the annealed MoS_2_ indicates defect generation. Inset: Integrated intensity plot of the LO(*M*) mode as a function of annealing temperature, ranging from 300 to 600 °C. The intensity increase of the defect mode with annealing temperature indicates an increase in the sulfur vacancy density. (c) XPS spectra of the Mo 3d core level in pristine and 600 °C Ar/H_2_ annealed MoS_2_. This measurement was performed by using synchrotron 1 keV soft X-rays. Pristine MoS_2_ shows the characteristic doublet from stoichiometric MoS_2_ (denoted in red) and S 2s peak (denoted in green). The annealed MoS_2_ shows a second Mo 3d doublet from nonstoichiometric MoS_*x*_ (0 < *x* < 2, denoted in blue), indicating defect generation. Inset: Optical microscopy image of monolayer MoS_2_ that is grounded with an In/Au electrode during XPS measurements. Scale bar = 20 μm.

We prepared monolayer MoS_2_ samples on SiO_2_ substrates by mechanical exfoliation and CVD growth (see the “Experimental Methods” section for details). To generate sulfur vacancies, the samples were annealed in an Ar/H_2_ (95%/5%) atmosphere for 30 min (Figure 1a) at temperatures ranging from 300 to 600 °C. We observed a clear change in optical contrast due to material degradation above 600 °C (Supporting Information (SI), Figure S1c). The Raman spectrum of pristine monolayer MoS_2_ in Figure 1b shows that it consists of a characteristic E^1^_2g_ in-plane mode peak at ∼385 cm^–1^ and A_1g_ out-of-plane mode peak at ∼405 cm^–1^.^31^ The weak satellite peak at ∼380 cm^–1^ is generally assigned to a longitudinal optical branch (LO(*M*)) due to sulfur vacancies.^32,33^ The intensity of the defect mode was found to increase with annealing from 300–600 °C, indicating that sulfur vacancy density gradually increased with temperature (Figure 1b, inset; see details in SI, Figure S1a). The most intense LO(*M*) defect mode was found in 600 °C annealed MoS_2_, indicating that under this condition, the sample contains the highest defect density.

Defect generation in the 600 °C annealed sample was corroborated by chemical analysis using synchrotron X-ray photoelectron spectroscopy (XPS) also termed photoemission spectroscopy (PES) (Figure 1c). The exfoliated monolayer flakes (lateral size of ∼20 μm, similar to the beam spot size) were grounded using lithographically patterned In/Au electrodes^34^ to avoid charging during XPS. To exclude the influence of potential defect generation by synchrotron X-ray radiation, we performed radiation damage tests using synchrotron 3 keV hard X-rays. No obvious changes in the XPS spectra were observed over several measurements at a fixed sample position (SI, Figure S2), indicating that beam-induced defects during measurements can be neglected.

XPS spectra of the Mo 3d doublet were measured using 1 keV soft X-rays as a surface-sensitive probe. Pristine MoS_2_ shows the characteristic doublet corresponding to binding energy of stoichiometric MoS_2_ at 230.4 eV (Mo 3d_5/2_)^35^ (see the Supporting Information for details of XPS fitting procedure). The annealed MoS_2_ shows the doublet of stoichiometric MoS_2_ at 231.0 eV (Mo 3d_5/2_), as well as peaks from nonstoichiometric MoS_*x*_ (0 < *x* < 2) at 230.1 eV (Mo 3d_5/2_), respectively. The defect concentration for both pristine and annealed MoS_2_ is extracted from stoichiometry calculation, using the core level spectra of Mo 3d and S 2s. The actual stoichiometry is given by
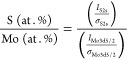
Here, S (at.%) and Mo (at.%) are the atomic percent (at. %) of S and Mo, *I*_S2s_ and *I*_Mo3d5/2_ are the integrated intensity of S 2s peak and Mo 3d_5/2_ peak. σ_S2s_ and σ_Mo3d5/2_ are the photoionization cross-section at photon energy of 1 keV modeled by Scofield, where σ_S2s_ = 0.44168, and σ_Mo3d5/2_ = 2.4679.^36^ For pristine MoS_2_, the actual stoichiometry is calculated to be MoS_1.99_, which leads to a defect concentration of 0.5%. In a perfect superstructure of 1H MoS_2_, the sulfur–sulfur distance is 3.162 Å,^37^ thus the density of S atoms in monolayer MoS_2_ can be calculated to be ∼2.3 × 10^15^ cm^–2^. For a defect concentration of 0.5%, the defect density is approximated to be ∼1.15 × 10^13^ cm^–2^, similar to reported values on pristine samples.^8^ For the annealed MoS_2_, the calculated stoichiometry is MoS_1.84_ with a defect concentration of ∼7.7%, equivalent to a sulfur vacancy density of ∼1.8 × 10^14^ cm^–2^. It is worth noting that for the defective sample, the Mo 3d doublet is shifted to higher binding energies by 0.6 eV (as indicated by the dashed vertical red line in Figure 1c), indicating electron doping mediated by sulfur vacancies—consistent with donor states detected by scanning tunneling microscopy/spectroscopy^38^ and deep level transient spectroscopy (DLTS).^39^

**Figure 2:**
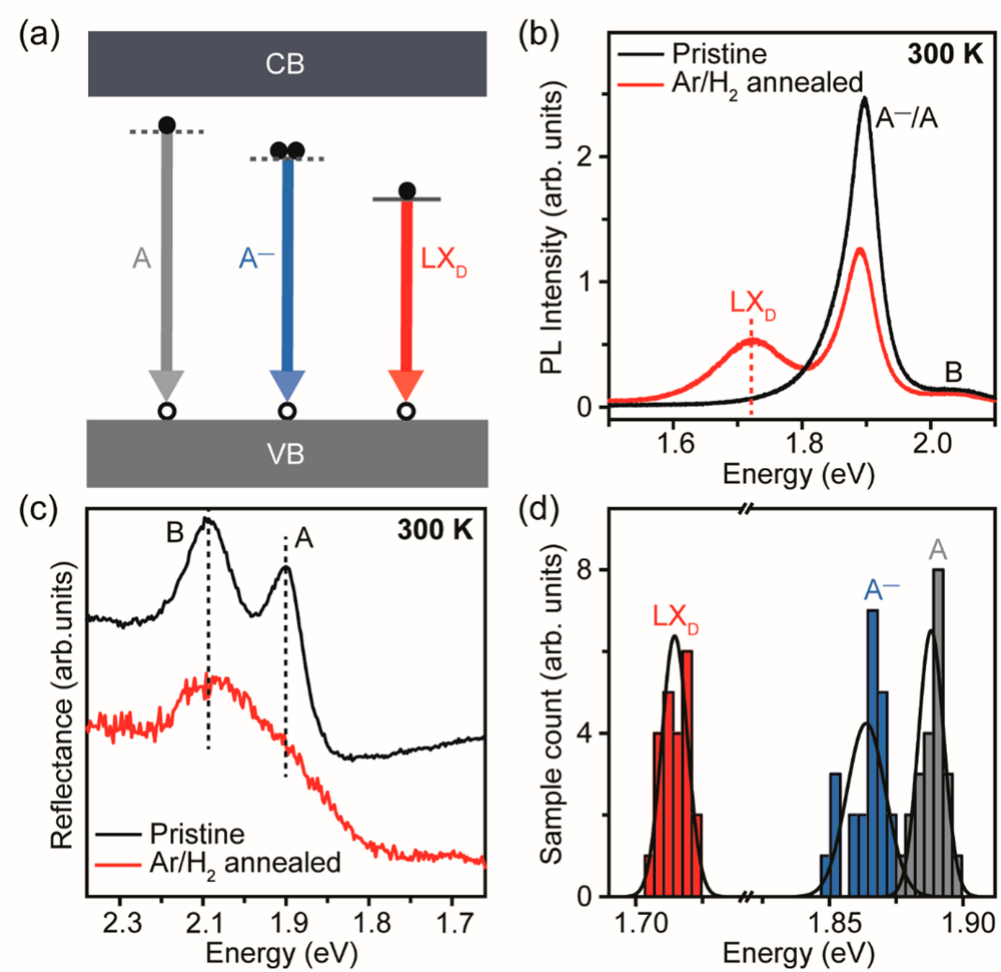
RT optical properties induced by sulfur vacancies in monolayer MoS_2_. (a) Proposed schematic energy band diagram of the sulfur-deficient monolayer MoS_2_. The gray arrow (neutral exciton A) represents an electron in the conduction band (CB) recombining with a hole in the valence band (VB). The blue arrow (negative trion A^–^) represents the recombination of two electrons and a hole. The red arrow (LX_D_) represents a localized electron recombining with a hole. The horizontal dashed lines represent virtual energy states, while the solid horizontal line represents real energy states due to defects. (b) RT PL spectra of pristine and 600 °C Ar/H_2_ annealed monolayer MoS_2_. Besides the A^–^ trion/A exciton (A^–^/A) and B exciton emission shown in the pristine MoS_2_ spectrum, the annealed MoS_2_ spectrum shows a low-energy localized emission LX_D_ mediated by defects, as indicated by the dashed vertical red line. (c) Reflectance spectra of pristine and 600 °C Ar/H_2_ annealed monolayer MoS_2_. Pristine MoS_2_ spectrum indicates the reflectance signals of the A exciton and B exciton. The broadening reflectance spectrum of the annealed MoS_2_ indicates the generation of in-gap defect states. (d) Statistical histogram of emission energy of A, A^–^, and LX_D_ emission for 22 individual 600 °C Ar/H_2_ annealed samples showing the high consistency and reproducibility of the LX_D_ emission.

The defects generated through annealing lead to the formation of electronic in-gap states in monolayer MoS_2_, as shown in the schematic energy band diagram in Figure 2a—consistent with the band structure of sulfur-deficient monolayer MoS_2_ by density functional theory (DFT) calculations.^40^ We have used PL spectroscopy (Figure 2b; see details in SI, Figure S1b) and reflectance measurements (Figure 2c) to characterize the RT optical properties of monolayer MoS_2_. In pristine MoS_2_, the RT PL spectrum shows the characteristic A^–^ trion/A exciton (A^–^/A) peak at 1.90 eV and B exciton peak at 2.05 eV. The reflectance spectrum shows corresponding A (1.91 eV) and B (2.06 eV) excitonic transitions.^41^ For the annealed sample, the RT PL spectrum shows a low-energy localized emission peak, LX_D_, that originates from defect-induced in-gap states with a large binding energy of 185 meV. The LX_D_ emission results from excitonic transitions between one unoccupied, in-gap defect state lying ∼0.6 eV below the conduction band minimum (CBM) and the other energy state at the valence band maximum (VBM).^40^ For monolayer MoS_2_, the binding energy of A exciton is ∼0.41 eV. Therefore, the binding energy of defect-mediated emission can be approximated to be ∼190 meV, which matches the binding energy of 185 meV of LX_D_ peak in our PL results. The reflectance spectrum is also broadened in the defective sample. The RT PL spectra were deconvoluted to extract the ratio of integrated intensity of A^–^ trion to A exciton, *I*(A^–^)/*I*(A) (SI, Figure S3a,b).^42^ The ratio was found to increase from 0.44 in pristine MoS_2_ to 1.06 in annealed MoS_2_, indicating increased electron doping from creation of defects, consistent with the XPS peak shift shown in Figure 1c.

The RT LX_D_ emission peak emerges weakly at 500 °C and becomes intense in 600 °C annealed samples (SI, Figure S1b). To understand the role of H_2_ in defect generation, we annealed monolayer MoS_2_ in an inert Ar-only atmosphere. The Ar-annealed samples did not show LX_D_ peaks at RT (see SI, Figure S4). We also confirm that the RT LX_D_ peak is not from the Ar/H_2_ annealed SiO_2_ substrate (see SI, Figure S5). Our results are reproducible and consistent across 22 samples measured in this study, as shown in the statistical histogram in Figure 2d (see the Supporting Information for details of the steady-state PL fitting procedure).

**Figure 3:**
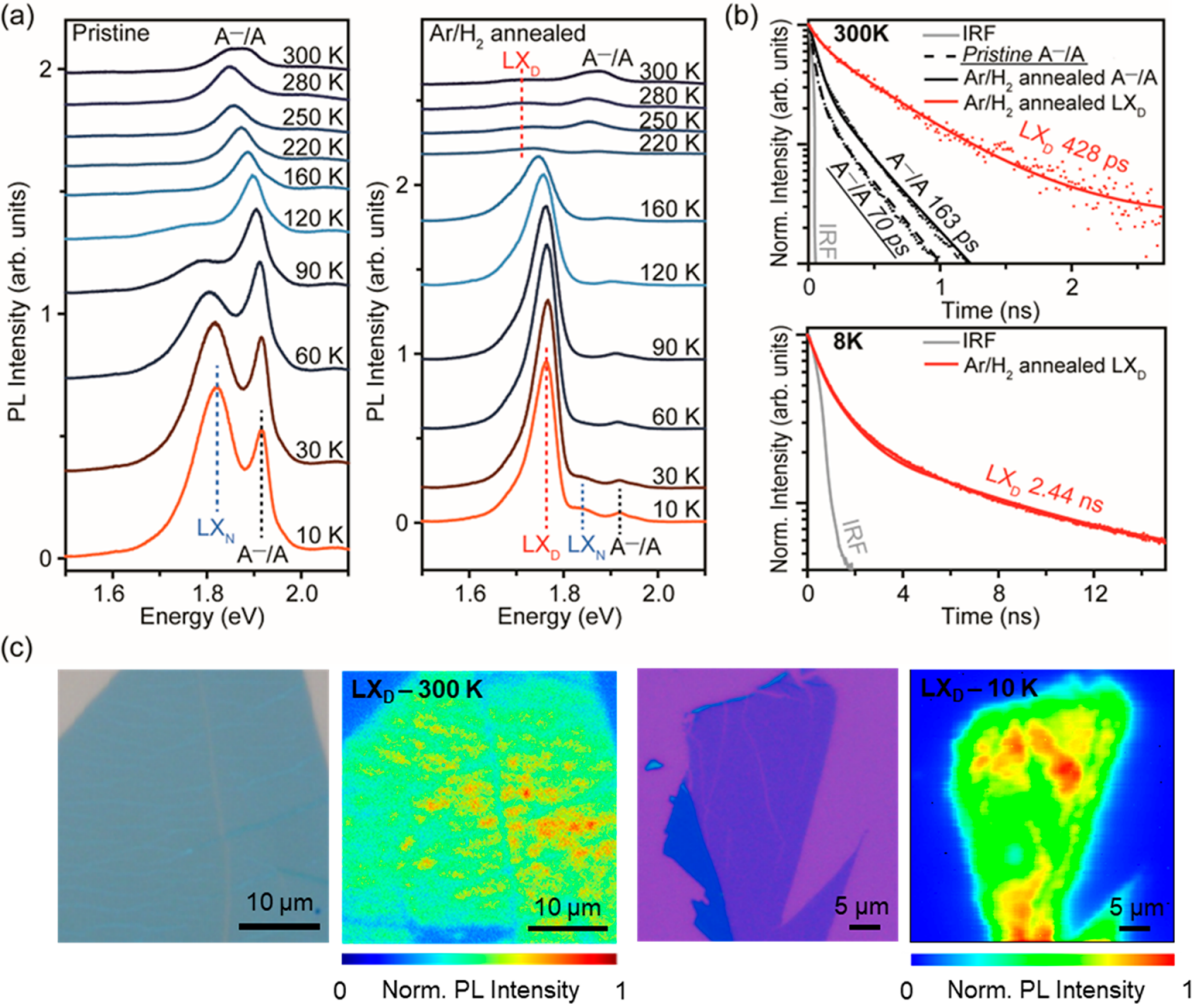
In-gap states induced by sulfur vacancies. (a) Temperature-dependent PL spectra of pristine and 600 °C Ar/H_2_ annealed monolayer MoS_2_. At 10 K, the LX_N_ peak is attributed to native absorbates, while the LX_D_ peak is attributed to sulfur vacancies. The continuous increase of LX_N_ and LX_D_ over the A^–^ trion/A exciton emission with decreasing measurement temperature indicates the temperature-dependent localization of in-gap states. (b) PL decays of pristine and 600 °C Ar/H_2_ annealed monolayer MoS_2_ measured at 300 and 8 K show longer lifetimes of localized excitons at in-gap states. (c) Intensity maps of LX_D_ emission measured at 300 and 10 K, with corresponding optical microscopy images of target flake, showing the distribution of LX_D_ emission over the monolayer.

To understand how the localization of excitons at defect states is related to the evolution of the LX_D_ peak, we performed temperature-dependent PL (Figure 3a). For pristine MoS_2_ at 10 K, the most intense peak is the LX_N_ peak, which is generally attributed to native absorbates^13^ accompanied by a weakened A^–^/A peak. In contrast, for the annealed MoS_2_, the most intense emission peak is the LX_D_ peak, with the LX_N_ and A^–^/A peaks significantly suppressed. The emission energy of LX_D_ is consistent with that of sulfur-vacancy-induced emission generated by *in vacuo* annealing,^13^ focused He^+^ irradiation,^13^ and proton irradiation.^30^ As the samples were cooled from 300 to 10 K, the dominant recombination pathway shows a transition from A^–^/A to LX_N_ emission in the case of pristine MoS_2_. For the annealed sample, the transition from A^–^/A to LX_D_ emission indicates a strong localization of excitons at lower-lying in-gap states. The absorbates-related emission can be suppressed by full encapsulation in hexagonal boron nitride, as shown in SI, Figure S6.^43−45^

In-gap states trap excitons and subsequently increase their PL decay lifetime.^29,30^ We measured the PL decay of the LX_D_ emission at 300 and 8 K by time-resolved PL spectroscopy (Figure 3b). At 300 K (Figure 3b, top), pristine MoS_2_ shows an A^–^/A exciton lifetime of 70 ps, and the annealed MoS_2_ shows a slightly longer A^–^/A exciton lifetime of 163 ps due to the increased electron doping, which results in a larger proportion of trions^46^ (see the Supporting Information for details of the time-resolved PL decay fitting procedure). The LX_D_ exciton lifetime was measured to be longer, 428 ps. When the sample was cooled to 8 K, the A^–^/A exciton lifetime was below the detection limit of our instrument. In contrast, the LX_D_ exciton in annealed MoS_2_ was significantly long-lived, with a lifetime of 2.44 ns (Figure 3b, bottom). The long lifetimes of LX_D_ excitons support the idea that they are localized at defect sites. To probe the distribution of defects in the annealed MoS_2_, we obtained intensity maps of LX_D_ peak at 300 and 10 K (Figure 3c). The LX_D_ emission clearly shows consistent spectral characteristics with some variations in the intensity (SI, Figure S7).

**Figure 4:**
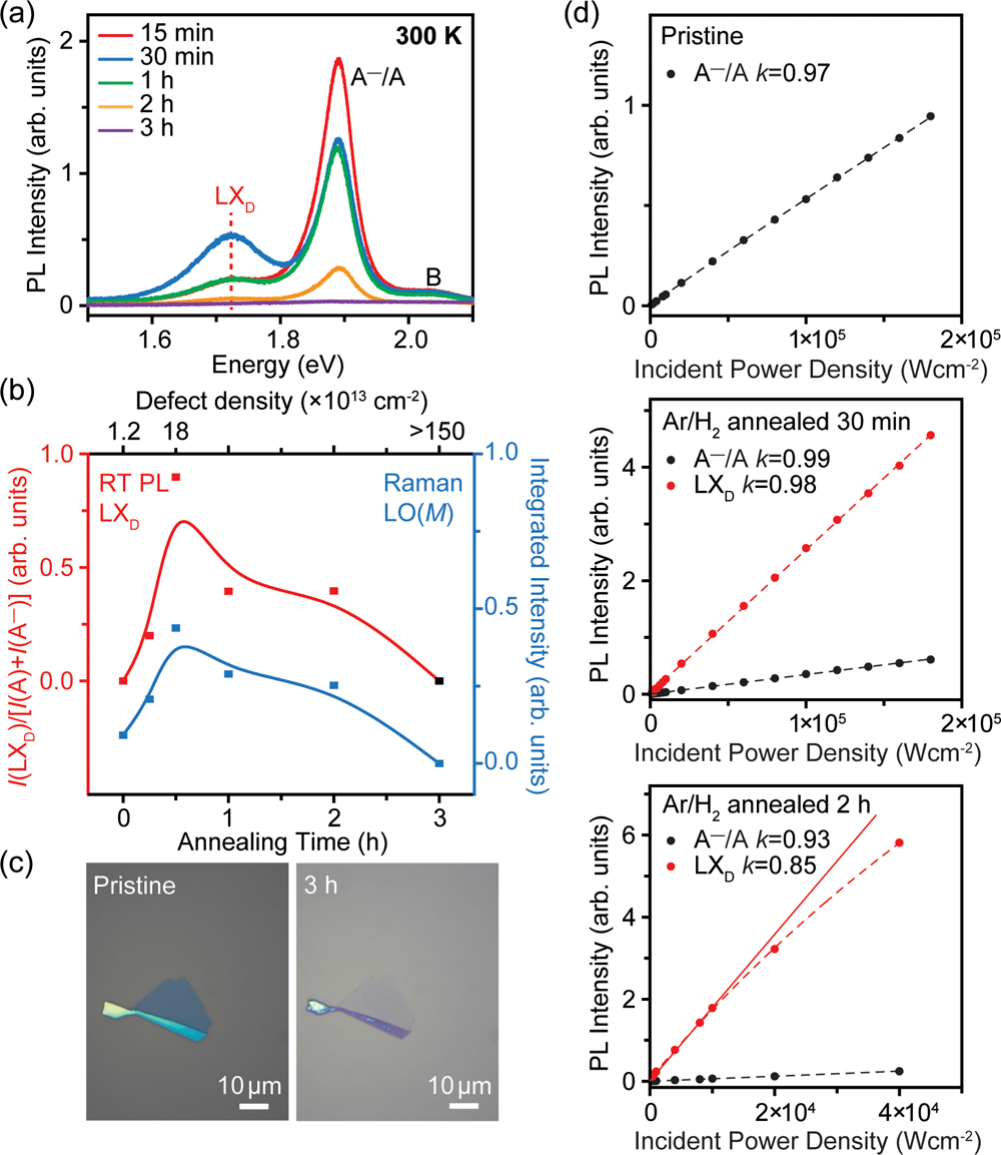
Evolution of monolayer MoS_2_ with increasing annealing time at 600 °C. (a) RT PL spectra of monolayer MoS_2_ with different annealing times. (b) (left) Evolution of integrated RT PL intensity ratio of LX_D_ emission to the sum of A exciton and A^–^ trion emission, referred to as *I*(LX_D_)/[(*I*(A) + *I*(A^–^)]. (right) Evolution of the integrated Raman intensity of the LO(*M*) defect mode. The most intense RT LX_D_ emission and LO(*M*) mode are both observed in 30 min annealed MoS_2_. (c) Optical microscopy images of pristine and 3 h Ar/H_2_ annealed monolayer MoS_2_ show clear degradation of monolayer MoS_2_ through annealing. (d) Power-dependent PL spectra of pristine, 30 min, and 2 h Ar/H_2_ annealed monolayer MoS_2_ measured at 10 K. A^–^/A shows linear dependence, while LX_D_ shows linear dependence in 30 min annealed MoS_2_, whereas saturation dependence in 2 h annealed MoS_2_. The power exponent *k* is obtained from fitting of logarithmic plots, where *k* = ∼1 implies excitonic transition and *k* < 1 suggests defect-mediated recombination.

To investigate the evolution of LX_D_ emission with defect density, we characterized the RT LX_D_ emission as a function of the annealing time at 600 °C (Figure 4a,b). In pristine MoS_2_, in which the density of sulfur vacancies was ∼1.15 × 10^13^ cm^–2^, the LX_D_ defect peak was absent. As the annealing time was increased, the LX_D_ emission rose in intensity, reaching the maximum at 30 min, where the density of defects was ∼1.8 × 10^14^ cm^–2^, supported through XPS measurements. Increasing the annealing time beyond 30 min results in a decrease in intensity of all spectral features (A^–^/A and LX_D_ emission), which could be due to gradual material degradation at higher defect densities. Our previous work on sulfur vacancy generation using focused He^+^ irradiation (0.24 kJ/cm^2^) showed that at defect densities >1.5 × 10^15^ cm^–2^, the MoS_2_ lattice structure is damaged and the PL is quenched,^12^ similar to the sample annealed in Ar/H_2_ for 3 h (see Figure 4c). The dependence of the LX_D_ emission on defect concentration can be well correlated with the LO(*M*) defect mode in Raman spectra, and both reach their maximum intensity at an annealing time of 30 min (Figure 4b; see details in SI, Figure S8). Our results suggest that the strongest RT LX_D_ emission in 2D MoS_2_ occurs at a sulfur vacancy concentration of ∼1.8 × 10^14^ cm^–2^.

We performed power-dependent measurements at 10 K to study how the filling of defect states influences PL properties (Figure 4d). The PL intensity (*I*_PL_) follows a power-law dependence with excitation laser power (*L*_E_) so that *I*_PL_ ∝ *L*_E_^*k*^, where *k* is power exponent.^47^ Excitonic transitions typically exhibit linear excitation power dependence (*k* = ∼1), while transitions from localized excitons at lattice defects saturate at higher power values (*k* < 1) due to the finite availability of trap states.^48,49^ As shown in Figure 4d, pristine MoS_2_ shows a linear dependence of the A^–^/A peak (*k* = 0.97, see the logarithmic plot in SI, Figure S9). Similar linear dependence is observed in the A^–^/A peak for the 30 min annealed MoS_2_ (*k* = 0.99) and 2 h annealed MoS_2_ (*k* = 0.93). While LX_D_ shows a linear dependence in the 30 min annealed MoS_2_ (*k* = 0.98), the peak shows a clear sublinear behavior in the 2 h annealed MoS_2_ (*k* = 0.85), consistent with previous reports.^47,48^ The absence of saturation in the power dependence of LX_D_ for the 30 min annealed MoS_2_ suggests that this material may host more optically active defect sites for radiative recombination, which possibly requires higher excitation fluence to observe saturation. This in fact allows us to observe a clear PL emission from the defect states at RT, in contrast to previous observations of defect-related emission that have mostly been limited to low temperatures (SI, Table S1).

**Figure 5:**
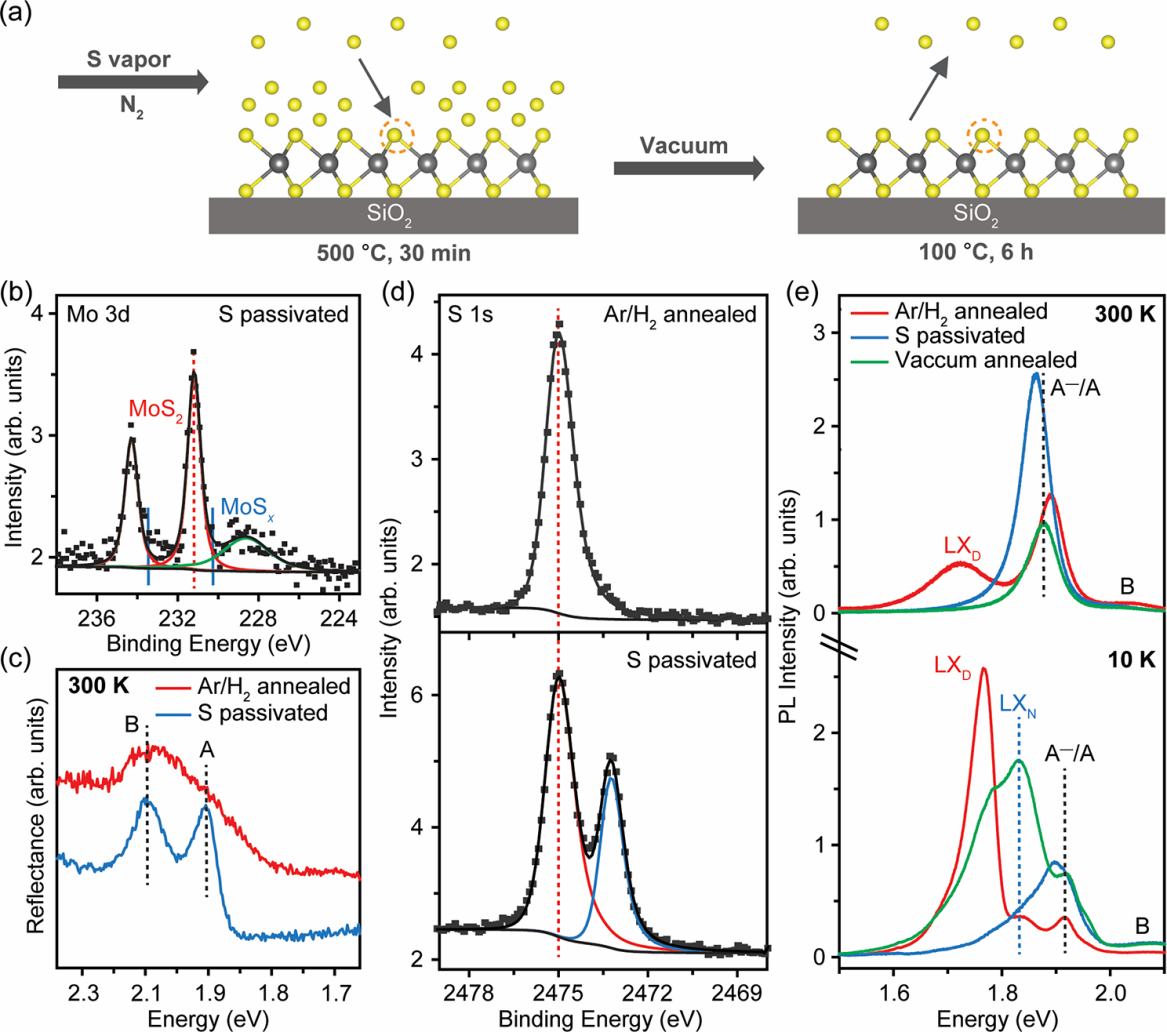
Sulfur passivation of Ar/H_2_ annealed (600 °C, 30 min) monolayer MoS_2_. (a) Schematic of annealing the Ar/H_2_ annealed (600 °C, 30 min) monolayer MoS_2_ in sulfur vapor. (b) XPS spectra of Mo 3d in sulfur passivated monolayer MoS_2_. The absence of nonstoichiometric MoS_*x*_ (denoted in blue) indicates the passivation of sulfur vacancies. (c) Reflectance spectra of sulfur passivated monolayer MoS_2_ restoring to pristine with the A and B excitonic edges recovered. (d) XPS spectra of S 1s in Ar/H_2_ annealed and sulfur passivated monolayer MoS_2_ probed by synchrotron 3 keV hard X-rays. The Ar/H_2_ annealed MoS_2_ only shows a pristine S 1s peak (denoted in red), whereas the passivated MoS_2_ shows two peaks. The second S 1s peak (denoted in blue) indicates the physisorbed sulfur on MoS_2_ surface. (e) PL spectra of Ar/H_2_ annealed (600 °C, 30 min), sulfur passivated, and vacuum annealed monolayer MoS_2_. At 300 K, the LX_D_ is absent in both sulfur passivated and vacuum annealed monolayer MoS_2_. At 10 K, the LX_D_ is still absent in sulfur passivated MoS_2_ whereas it weakly reappears in vacuum annealed MoS_2_.

The sulfur vacancies generated by annealing at 600 °C for 30 min in Ar/H_2_ give interesting PL properties but are typically detrimental to electronic transport. Thus, there is interest in passivating them to reduce carrier doping and scattering. We therefore investigated the filling of sulfur vacancies by exposing the defective sample to sulfur vapor at 500 °C (see schematic in Figure 5a). In the XPS spectra of the Mo 3d doublet (Figure 5b), we observe the absence of the nonstoichiometric MoS_*x*_ signal in the MoS_2_ samples annealed at 600 °C in Ar/H_2_ after annealing in sulfur vapor. The actual stoichiometry after sulfur vapor treatment was found to be MoS_2.05_, comparable to that of the pristine MoS_2_ samples. The reflectance spectrum of the passivated sample shown in Figure 5c is similar to that of pristine MoS_2_, with the A and B excitonic edges recovered. In PL spectra at 300 and 10 K, the LX_D_ peak is absent for the sulfur passivated sample (Figure 5e). However, XPS results reveal an unusual shift of the Mo 3d doublet in sulfur passivated MoS_2_ to higher binding energies of 231.2 eV (Mo 3d_5/2_), which indicates significant electron doping. This can be correlated with the RT PL spectrum, which shows a higher *I*(A^–^)/*I*(A) peak ratio of 2.05 (SI, Figure S3c). The electron doping is likely due to excessive physisorbed sulfur on the surface of MoS_2_ during passivation. To confirm this, we measured synchrotron XPS S 1s spectra (Figure 5d), which reveal that Ar/H_2_ annealed MoS_2_ only shows a pristine S 1s peak at 2475.0 eV, whereas passivated MoS_2_ shows two peaks (see the Supporting Information for details of the XPS fitting procedure). The second peak with a lower binding energy of 2473.2 eV can be attributed to physisorbed sulfur on the MoS_2_ surface.^50^

We sought to remove the weakly physisorbed sulfur on MoS_2_ by annealing the passivated sample at 100 °C under a high vacuum. We chose 100 °C because it is sufficient for subliming unbonded sulfur, but not high enough to create new vacancies.^13^ The PL spectra in Figure 5e show that the A^–^/A emission peak is blue-shifted close to the value measured for pristine MoS_2_. Furthermore, the *I*(A^–^)/*I*(A) ratio was decreased to 0.19, indicating that electron doping is largely reduced (SI, Figure S3d). At 10 K, the defect-mediated LX_D_ emission reappears, albeit with lower intensity than absorbate-related LX_N_ emission, showing that the passivation of in-gap states by annealing in sulfur vapor is a combination of chemical bonding and electron doping. These results show that it is possible to controllably engineer defects and passivate them in both exfoliated and CVD-grown MoS_2_ (SI, Figure S10). Having optimized these protocols for MoS_2_, we extended them to other TMDs such as WS_2_, MoSe_2_, and WSe_2_, as shown in SI, Figure S11. Similar RT defect peaks were observed in monolayer WS_2_, while no RT defect peak was observed in selenide monolayer TMDs, including MoSe_2_ and WSe_2_.

## Conclusion

We have demonstrated that by controlling the temperature, duration, and environment of thermal annealing, we can reproducibly generate sulfur vacancies in 2D MoS_2_. The sulfur vacancies induce in-gap electronic states and lead to RT localized exciton emission that can be tuned by the annealing conditions. We also show that sulfur vacancies can be passivated by annealing in sulfur vapor. Our results provide a simple technique to reproducibly create chalcogen vacancies in wafer-scale TMD films, which may enable functional devices.

## Experimental Methods

### Sample Preparation

#### Mechanical Exfoliation

Monolayer TMDs, including MoS_2_, WS_2_, MoSe_2_, and WSe_2_, were prepared by mechanical exfoliation from commercial bulk crystal purchased from 2D Semiconductor using a polydimethylsiloxane (PDMS)-assisted exfoliation method. Target flakes were dry-transferred onto SiO_2_/Si substrate and put in a 1 in. tube furnace. The samples were flowed with 450 sccm of N_2_ (99.9999% purity) for 10 min to remove tube oxygen and then heated to 250 °C for 1 h under 100 sccm of N_2_ to clean contaminants.

#### CVD Growth

Monolayer MoS_2_ was grown by CVD using MoO_3_ and sulfur powders as precursors. NaOH was dissolved in water with a concentration of 4 mg/mL and spin-coated on SiO_2_/Si wafers as a primer. Then 0.125 mg of MoO_3_ and 30 mg of sulfur were placed in two alumina boats, respectively, in the center and upstream of the tube furnace. SiO_2_/Si wafers were placed face-down on top of MoO_3_. The furnace was heated to 700 °C and maintained for 10 min for the growth of MoS_2_ layers. A 260/60 sccm of N_2_ was used as carrier gas. Then the furnace was cooled down to RT, and samples are collected.

### Ar/H_2_ Annealing

Samples were flowed with 450 sccm Ar/H_2_ (95%:5%) for 10 min to remove tube oxygen and heated to 200 °C under 70 sccm Ar/H_2_ for 10 min to remove tube moisture. Then samples were heated to the target annealing temperature and kept there for the target time.

### Annealing in Sulfur Vapor for Passivation

First, 8 mg of sulfur powders were put on the upstream of tube furnace and Ar/H_2_ annealed samples were put in the center of furnace. Then the tube was flowed with 450 sccm of N_2_ for 10 min to remove tube oxygen and then heated to the target annealing temperature under 50 sccm of N_2_ and kept for 30 min.

### Vacuum Annealing

Passivated samples were evacuated to <10^–6^ mbar and then heated to 100 °C for 6 h.

After each annealing, the furnace was rapidly cooled back to RT, and samples were removed from the tube for measurement.

### Measurement

Optical images of samples were taken on a Nikon Eclipsec LV150N optical microscope.

RT Raman and steady-state PL spectra were collected on a Renishaw inVia confocal microscope using 532 nm laser excitation. RT PL images and time-resolved PL measurements were taken on a custom-built optical microscope setup. Excitation of 532 nm was provided. The wide-field image was acquired using a 60× objective on an EMCCD camera, and the PL lifetimes were measured using TCSPC electronics after filtering out using selective band-pass filter. Reflection microscopy was performed with the same setup for PL measurement, coupled to a monochromator. Images from variable wavelength excitation were provided in reflection geometry on a camera.

Cryogenic PL spectra, PL mapping, and time-resolved PL measurements were measured on a Kymera spectrometer and Janis (ST-500) cryostat with a closed-cycle helium refrigerator. Continuous wave 474 nm laser and pulsed 405 nm laser were used as excitations for PL spectra/mapping and time-resolved PL measurements, respectively. Long pass filters and tunable bandpass filters were used to select the peaks during time-resolved PL measurements.

XPS measurements were performed at the I09 beamline at Diamond Light Source, UK. Both soft (100–2100 eV) and hard (2.1–20 keV) X-rays were directed to the same spot on the sample. The beam size was around 15 μm by 35 μm. The core level spectra of MoS_2_ were acquired at photon energy of 1 keV and S 1s spectra were collected with excitation energy of 3 keV. Samples were heated at 80 °C under 10^–7^ mbar for 1 h to remove physisorbed species prior to loading into measurement chamber (10^–10^ mbar). The Fermi level was calibrated by using a clean Au foil in contact with the sample holder. The spectral resolution was determined by measuring and fitting the Fermi edge of a Au foil with a Gaussian broadened Fermi–Dirac distribution and was determined to be 0.28 and 0.21 eV for the 3 and 1 keV measurements, respectively.

## Supporting Information Available

Raman spectra of monolayer MoS_2_ annealed through 300–600 °C Ar/H_2_ annealing; synchrotron X-ray radiation damage tests of monolayer MoS_2_; deconvoluted RT PL spectra of pristine, Ar/H_2_ annealed, sulfur passivated, and vacuum annealed monolayer MoS_2_; Raman spectra of monolayer MoS_2_ annealed through 300–550 °C Ar-only annealing; comparison of RT PL spectrum of monolayer MoS_2_ on pristine and 600 °C Ar/H_2_ annealed SiO_2_ substrate; 10 K PL spectra of 600 °C Ar/H_2_ annealed monolayer MoS_2_ with half and full hBN encapsulation; 10 K LX_D_ intensity map of 600 °C Ar/H_2_ annealed monolayer MoS_2_; Raman spectra of 600 °C Ar/H_2_ annealed MoS_2_ with different annealing times; power-dependent PL spectra of pristine, 30 min, and 2 h Ar/H_2_ annealed monolayer MoS_2_ measured at 10 K; RT PL spectra of CVD-grown monolayer MoS_2_ with defect generation and passivation; RT PL spectra of monolayer WS_2_, MoSe_2_, and WSe_2_ through Ar/H_2_ annealing with different annealing temperatures and times (PDF)
