# Supplementary material for: Room-Temperature Photoluminescence Mediated by Sulfur Vacancies in 2D Molybdenum Disulfide
Source: ACS Nano. 2023 Jul 7;17(14):13545–53. doi: 10.1021/acsnano.3c02103 (PMC10373523; doi:10.1021/acsnano.3c02103)
Supplement: Supplementary file 1 — nn3c02103_si_001.pdf [file nn3c02103_si_001.pdf]

Supporting Information
for
Room temperature photoluminescence mediated by sulfur vacancies in 2D molybdenum disulfide

Yiru Zhu, Juhwan Lim, Zhepeng Zhang, Yan Wang,* Soumya Sarkar, Hugh Ramsden, Yang Li, Han Yan, Dibya Phuyal, Nicolas Gauriot, Akshay Rao, Robert L. Z. Hoye, Goki Eda, Manish Chhowalla*

Synchrotron XPS peak fitting procedure: All XPS results included in our work were fitted with Casa XPS software, using a Shirley background. The lineshape LA(2,1.2,100) was obtained by fitting the standard Au 4f spectrum. Referring to XPS fitting database, each Mo $3d_{3/2}$ – $3d_{5/2}$ doublet was constrained with position separation of 3.1 eV, peak area ratio of 2:3 (Mo $3d_{3/2}$: $3d_{5/2}$), and FWHM to be equal. No constraint is used for S 2s and S 1s peaks. The chi-square was always below 3.5.

Steady-state PL fitting procedure: Lorenzian components were fitted to the data using the Lumispy and Hyperspy Python libraries, (1) Peña, F. de la; Prestat, E.; Fauske, V. T.; Burdet, P.; Lähnemann, J.; Jokubauskas, P.; Furnival, T.; Nord, M.; Ostasevicius, T.; MacArthur, K. E.; Johnstone, D. N.; Sarahan, M.; Taillon, J.; Aarholt, T.; pquinn-dls; Migunov, V.; Eljarrat, A.; Caron, J.; Francis, C.; Nemoto, T.; Poon, T.; Mazzucco, S.; actions-user; Tappy, N.; Cautaerts, N.;

Somnath, S.; Slater, T.; Walls, M.; Winkler, F.; Ånes, H. W. Hyperspy/Hyperspy: Release v1.7.3, 2022. https://doi.org/10.5281/zenodo.7263263.

(2) Lähnemann, J.; Orri, J. F.; Prestat, E.; Johnstone, D. N.; Tappy, N. LumiSpy/Lumispy: Release v0.2, 2022. https://doi.org/10.5281/zenodo.6506534.

Time-resolved PL decay fitting procedure: All PL decays were fitted with a bi-exponential decay function *ExpDecay 2*: $y = y_0 + A_1 e^{-(x-x_0)/\tau_1} + A_2 e^{-(x-x_0)/\tau_2}$, where A_1 and A_2 are amplitude constants, τ_1 and τ_2 are time constants. The amplitude-weighted average lifetimes were calculated by: $\tau_{average} = \frac{A_1\tau_1 + A_2\tau_2}{A_1 + A_2}$.

	Sample	PL peak	$\tau_{average}$ (ns)	A_1	τ_1 (ns)	A_2	τ_2 (ns)
**300 K**	Pristine	A^{-}/A	0.070	3.005	0.047	0.272	0.322
	Ar/H_2 annealed	A^{-}/A	0.163	0.687	0.075	0.323	0.351
		LX_D	0.428	0.284	0.094	0.695	0.565
**8 K**	Pristine	A^{-}/A	Below the detection limit				
	Ar/H_2 annealed	A^{-}/A					
		LX_D	2.442	0.757	0.946	0.231	7.348

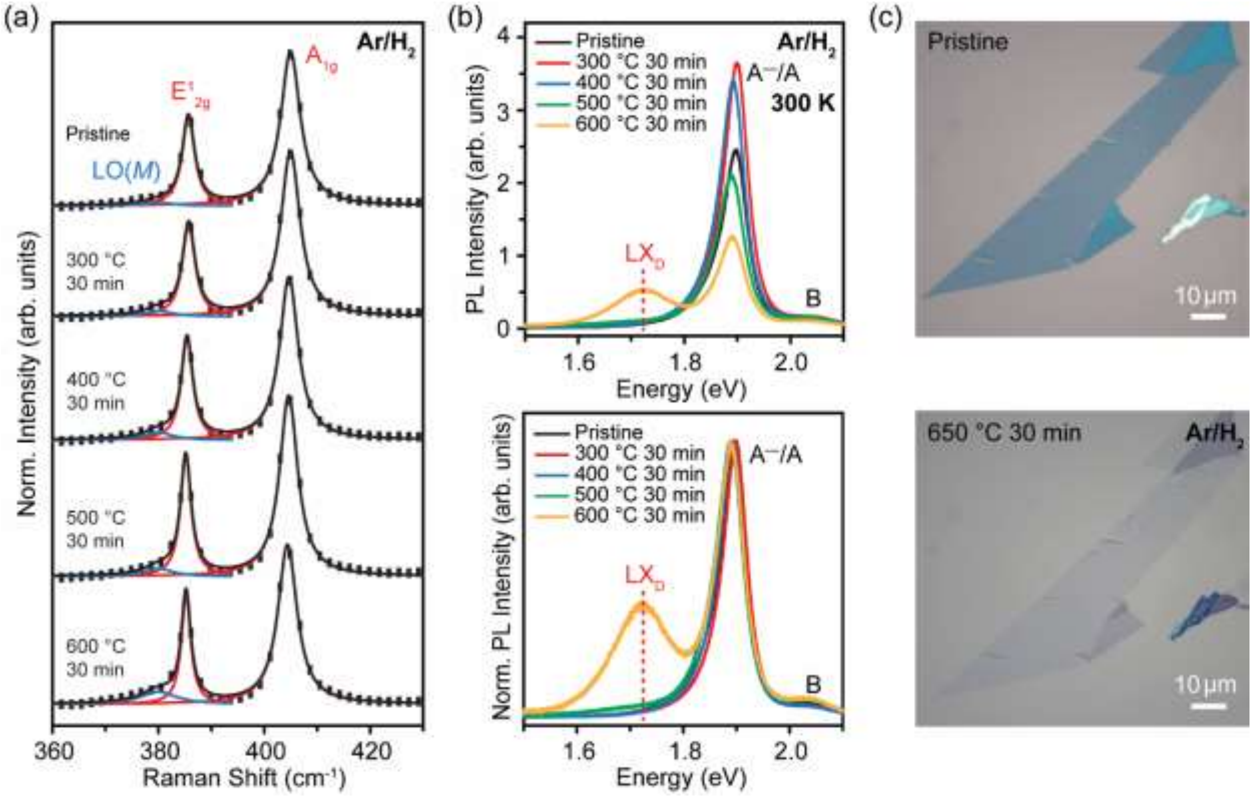

Figure S1. Evolution of monolayer MoS_2 annealed in an Ar/H_2 (95%/5%) atmosphere. (a) Raman spectra of monolayer MoS_2 through 300 – 600 °C Ar/H_2 annealing, normalized to the intensity of Si reference peak. The continuous increase of the normalized intensity of LO(*M*) defect mode (denoted in blue) with temperature, indicates mild defect generation through ≤600 °C Ar/H_2 annealing. (b) RT PL spectra of monolayer MoS_2 through 300 – 600 °C Ar/H_2 annealing showing LX_D with ≥500 °C Ar/H_2 annealing. Normalized PL spectra is normalized to the intensity of A^-/A peak. (c) Optical microscopy images of pristine and 650 °C Ar/H_2 annealed monolayer MoS_2 showing clear material degradation of monolayer flake.

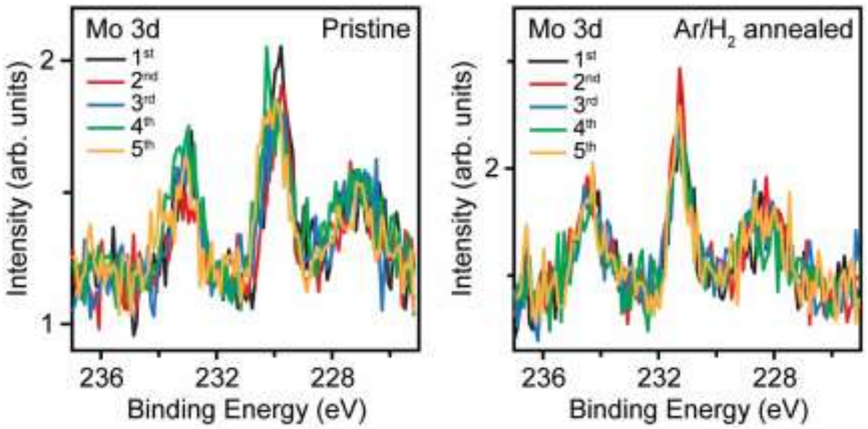

Figure S2. Synchrotron X-ray radiation damage tests of pristine and 600 °C Ar/H_2 annealed monolayer MoS_2 using synchrotron 3 keV hard X-rays. No obvious damages in MoS_2 are observed during several measurements at a fixed sample position.

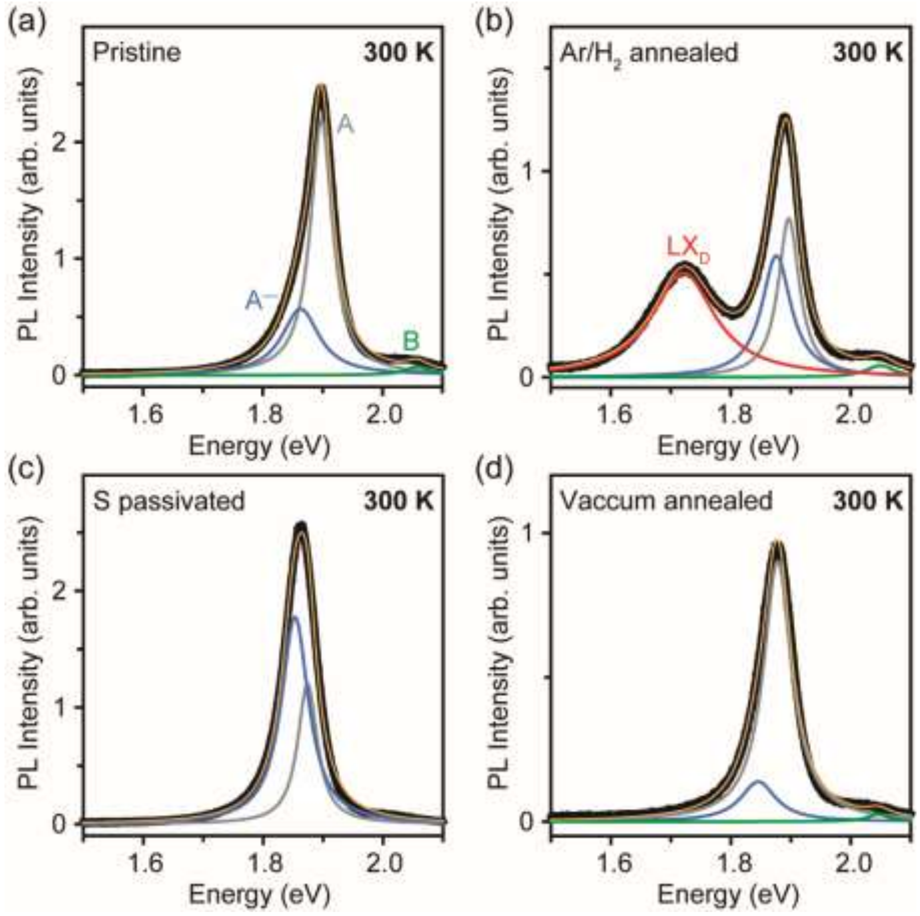

Figure S3. RT PL spectra of (a) pristine, (b) Ar/H_2 annealed, (c) sulfur passivated, (d) vacuum annealed monolayer MoS_2, fitted with Lorentz peaks. Denotation: A exciton in gray, A^{-} trion in blue, B exciton in green, LX_D emission in red, and cumulative fit curve in yellow.

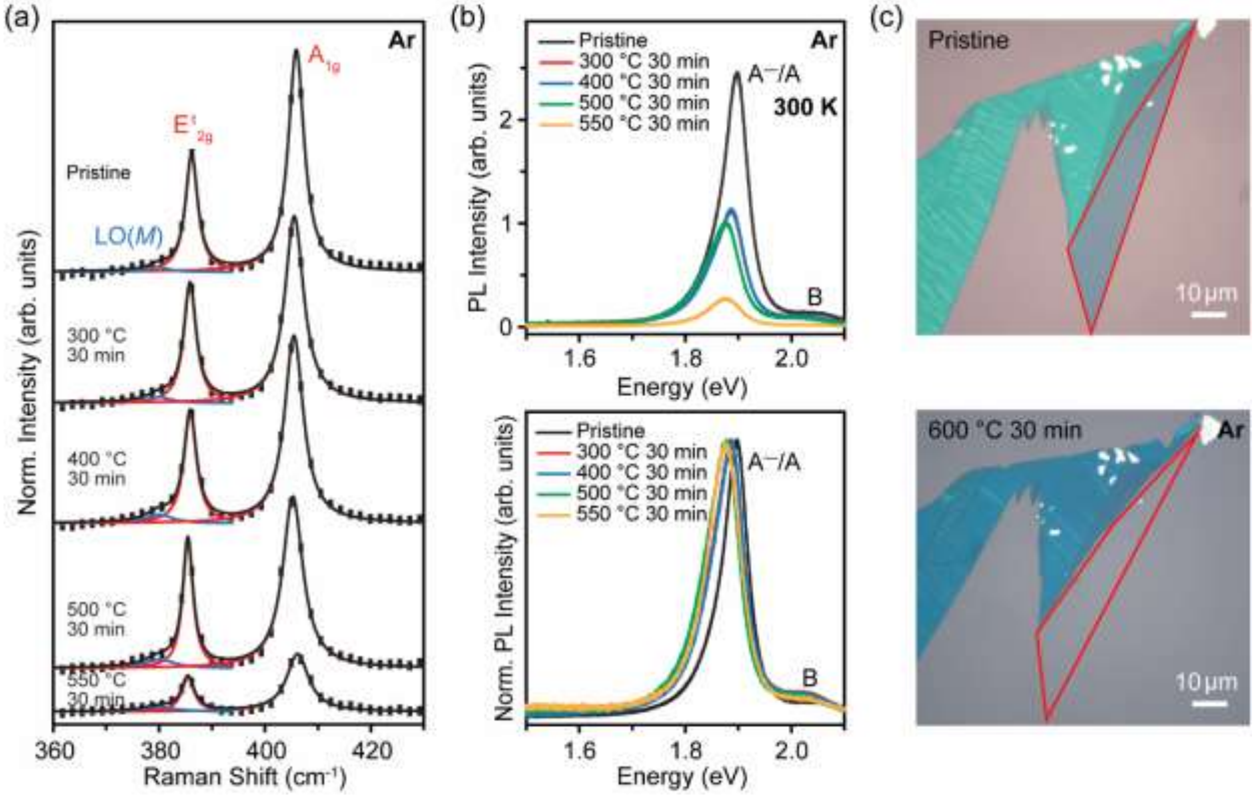

Figure S4. Evolution of monolayer MoS_2 annealed in an Ar-only atmosphere. (a) Raman spectra of monolayer MoS_2 through 300 – 550 °C Ar annealing, normalized to the intensity of Si reference peak. The normalized intensity of LO(*M*) defect mode (denoted in blue) slightly increases with ≤500 °C Ar annealing and then decreases with overall quenching through 550 °C Ar annealing, indicating mild to harsh defect generation. Normalized PL spectra is normalized to the intensity of A^{-}/A peak. (b) RT PL spectra of monolayer MoS_2 through 300 – 550 °C Ar annealing showing an absence of LX_D emission. (c) Optical microscopy images of pristine and 600 °C Ar annealed monolayer MoS_2 showing the material degradation of monolayer flake.

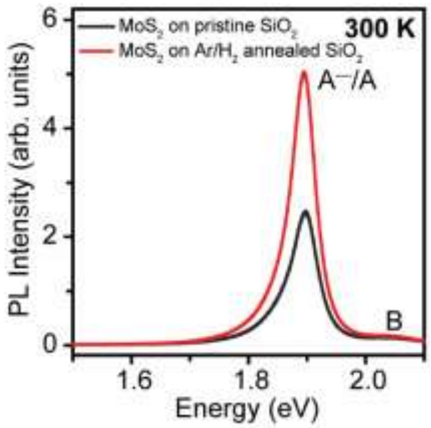

Figure S5. RT PL spectra of monolayer MoS_2 on pristine and 600 °C Ar/H_2 annealed SiO_2 substrate. No LX_D emission is observed in monolayer MoS_2 on 600 °C Ar/H_2 annealed SiO_2 substrate.

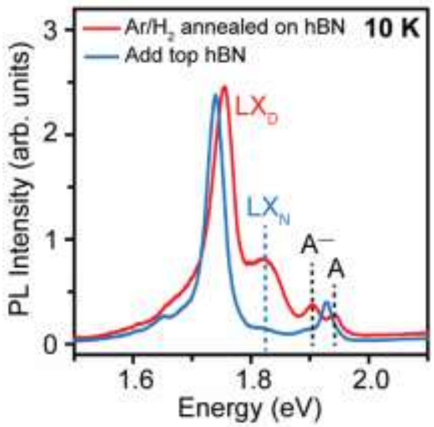

Figure S6. 10 K PL spectra of 600 °C Ar/H_2 annealed monolayer MoS_2 with half and full hBN encapsulation. The decrease of LX_N with full hBN encapsulation indicates the suppression of native absorbates.

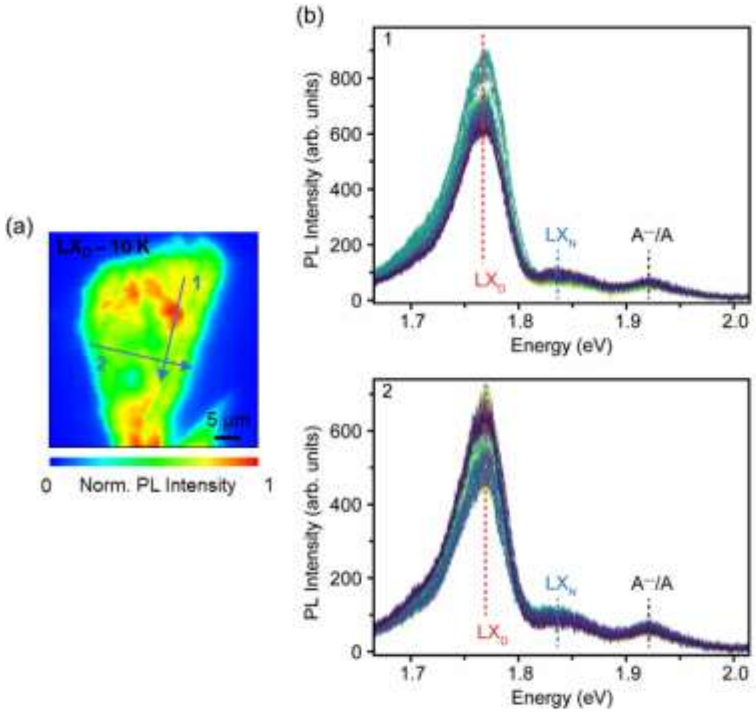

Figure S7. 10 K LX_D intensity map of 600 °C Ar/H_2 annealed monolayer MoS_2. (a) 10 K LX_D intensity map showing the distribution of LX_D emission over the monolayer. 36 points along blue arrow 1 and 37 points along blue arrow 2 are extracted, respectively. (b) PL spectra of 36 points along blue arrow 1 and 37 points along blue arrow 2 showing consistent spectral characteristics at each point .

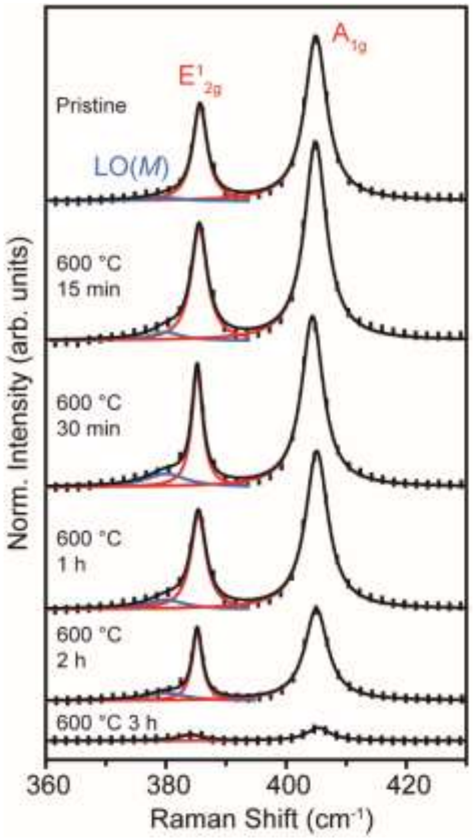

Figure S8. Evolution of Raman spectra of 600 °C Ar/H_2 annealed MoS_2 with different annealing times. The normalized intensity of LO(*M*) defect mode (denoted in blue) in Ar/H_2 annealed MoS_2 increases and reaches the maximum at 30 min, and then decreases with overall quenching.

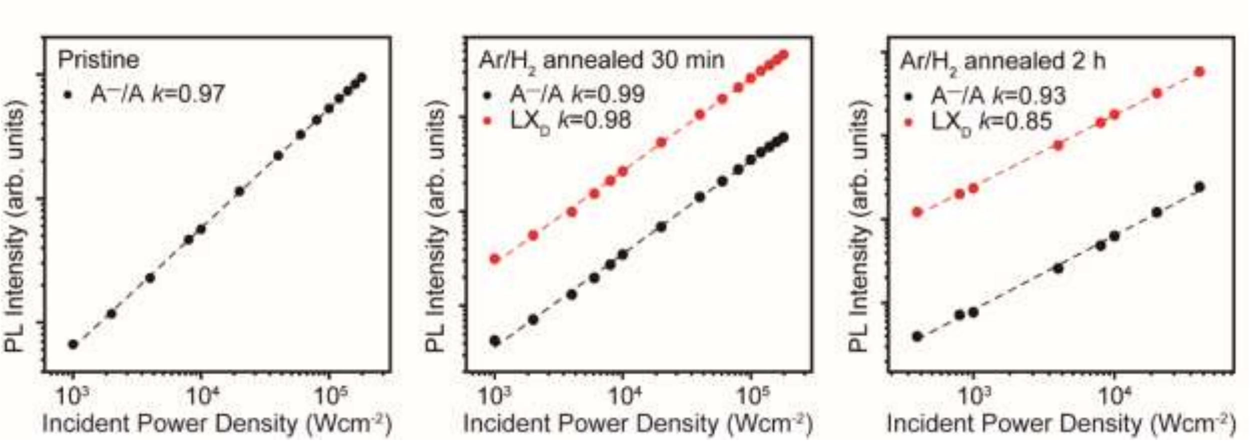

Figure S9. Logarithmic plot of power-dependent PL spectra of pristine, 30 min, and 2 h Ar/H_2 annealed monolayer MoS_2 measured at 10 K. A^{-}/A shows linear dependence, while LX_D shows linear dependence in 30 min annealed MoS_2 whereas sublinear dependence in 2 h annealed MoS_2. The power exponent k = ~1 implies excitonic transition and $k < 1$ suggests defect-mediated recombination.

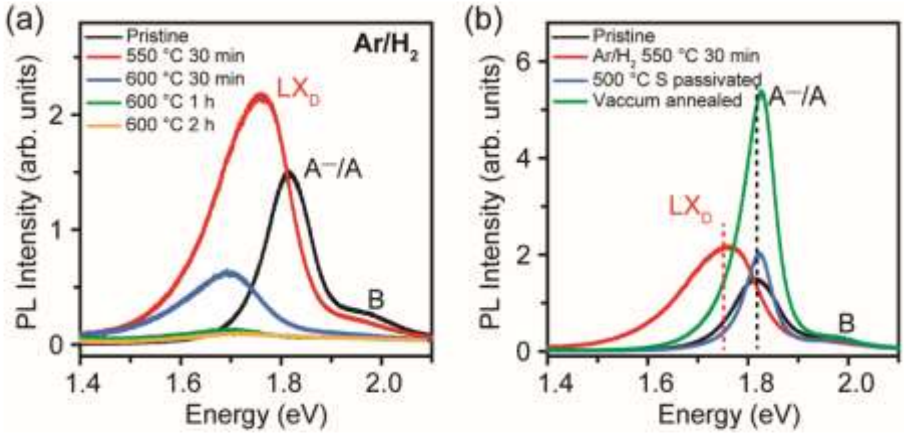

Figure S10. RT PL spectra of CVD-grown monolayer MoS_2 with defect generation and passivation. (a) RT PL spectra of CVD-grown monolayer MoS_2 with different Ar/H_2 annealing temperatures and times for defect generation. The PL red-shift of Ar/H_2 annealed MoS_2 indicates defect generation. (b) RT PL spectra of pristine, Ar/H_2 annealed, sulfur passivated, and vacuum annealed MoS_2. The restoration of PL to pristine indicates the passivation of sulfur vacancies.

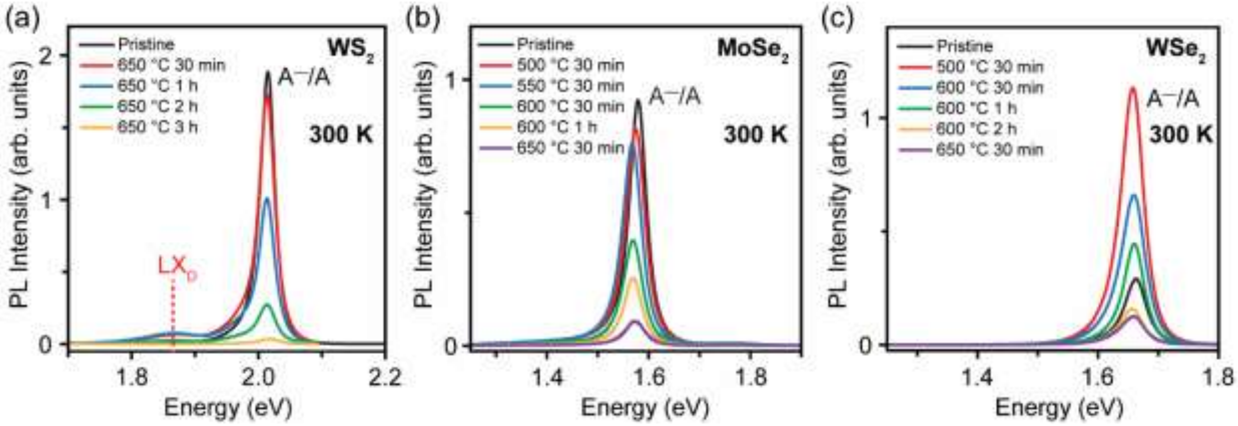

Figure S11. Evolution of RT PL spectra in other monolayer TMDs with different annealing temperatures and times. (a) RT PL spectra of monolayer WS_2 showing a RT defect peak LX_D at 1.867 eV. (b-c) RT PL spectra of monolayer $MoSe_2$ and WSe_2. No RT defect peaks are observed in selenide monolayer TMDs.

Table S1. Comparison between our work and published reports on defect-related optical emissions in monolayer TMDs.

Material	Treatment	Emission temperature	Ref.
MoS_2	X-ray irradiation	10 K	*ACS Nano* **2022**, *16*, 20364–20375.[1]
MoS_2	*In-vacuo* annealing	~20 K	*Nat. Commun.* **2021**, *12*, 1–8.[2]
MoS_2	Electron irradiation	No defect PL at RT	*npj 2D Mater. Appl.* **2022**, *6*, 31.[3]
MoS_2	Focused He ion irradiation	15 K	*ACS Photonics* **2021**, *8*, 669–677.[4]
MoS_2	H_2/He annealing	No defect PL at RT	*ACS Nano* **2022**, *16*, 6725–6733.[5]
MoS_2	Electron irradiation	Weak & broad PL at RT	*2D Materials* **2023**, *10*, 035002.[6]
MoS_2	Hydrogen plasma treatment	Weak & broad PL at RT	*Commun. Mater.* **2021**, *2,* 80.[7]
MoS_2	Focused He ion irradiation	Weak & broad PL at RT	*ACS Nano* **2019**, *13*, 9958–9964.[8]
WS_2	Focused He ion irradiation	10 K	*Appl. Phys. Lett.* **2022**, *121*, 183101.[9]
WS_2, WSe_2, MoS_2, $MoSe_2$	Proton irradiation	77 K	*Adv. Opt. Mater.* **2022**, *10*, 2201350.[10]
WS_2	Proton irradiation	4 K	*Nano Lett.* **2023**, *23*, 3754–3761.[11]
WSe_2	Electron irradiation & strain engineering	150 K	*Nat. Commun.* **2021**, *12*, 3585.[12]
MoS_2	Ar/H_2 annealing	RT	Our work

REFERENCES

(1) Grünleitner, T.; Henning, A.; Bissolo, M.; Zengerle, M.; Gregoratti, L.; Amati, M.; Zeller, P.; Eichhorn, J.; Stier, A. V.; Holleitner, A. W.; Finley, J. J.; Sharp, I. D. Real-Time Investigation of Sulfur Vacancy Generation and Passivation in Monolayer Molybdenum Disulfide via *in situ* X-Ray Photoelectron Spectromicroscopy. *ACS Nano* **2022**, *16*, 20364–20375.

(2) Mitterreiter, E.; Schuler, B.; Micevic, A.; Hernangómez-Pérez, D.; Barthelmi, K.; Cochrane, K. A.; Kiemle, J.; Sigger, F.; Klein, J.; Wong, E.; Barnard, E. S.; Watanabe, K.; Taniguchi, T.; Lorke, M.; Jahnke, F.; Finley, J. J.; Schwartzberg, A. M.; Qiu, D. Y.; Refaely-Abramson, S.; Holleitner, A. W.; Weber-Bargioni, A.; Kastl, C. The Role of Chalcogen Vacancies for Atomic Defect Emission in MoS_2. *Nat. Commun.* **2021**, *12*, 1–8.

(3) Wu, X.; Gu, Y.; Ge, R.; Serna, M. I.; Huang, Y.; Lee, J. C.; Akinwande, D. Electron Irradiation-Induced Defects for Reliability Improvement in Monolayer MoS_2-Based Conductive-Point Memory Devices. *Npj 2D Mater. Appl.* **2022**, *6*, 1–12.

(4) Klein, J.; Sigl, L.; Gyger, S.; Barthelmi, K.; Florian, M.; Rey, S.; Taniguchi, T.; Watanabe, K.; Jahnke, F.; Kastl, C.; Zwiller, V.; Jöns, K. D.; Müller, K.; Wurstbauer, U.; Finley, J. J.; Holleitner, A. W. Engineering the Luminescence and Generation of Individual Defect Emitters in Atomically Thin MoS_2. *ACS Photonics* **2021**, *8*, 669–677.

(5) Garcia-Esparza, A. T.; Park, S.; Abroshan, H.; Mellone, O. A. P.; Vinson, J.; Abraham, B.; Kim, T. R.; Nordlund, D.; Gallo, A.; Alonso-Mori, R.; Zheng, X.; Sokaras, D. Local Structure of Sulfur Vacancies on the Basal Plane of Monolayer MoS_2. *ACS Nano* **2022**, *16*, 6725–6733.

(6) Dash, A. K.; Swaminathan, H.; Berger, E.; Mondal, M.; Lehenkari, T.; Prasad, P. R.; Watanabe, K.; Taniguchi, T.; Komsa, H.-P.; Singh, A. Evidence of Defect Formation in Monolayer MoS_2 at Ultralow Accelerating Voltage Electron Irradiation. *2D Mater.* **2023**, *10*, 035002.

(7) Lee, J.-Y.; Kim, J. H.; Jung, Y.; Shin, J. C.; Lee, Y.; Kim, K.; Kim, N.; Zande, A. M. van der; Son, J.; Lee, G.-H. Evolution of Defect Formation during Atomically Precise Desulfurization of Monolayer MoS_2. *Commun. Mater.* **2021**, *2*, 1–10.

(8) Yang, J.; Wang, Y.; Lagos, M. J.; Manichev, V.; Fullon, R.; Song, X.; Voiry, D.; Chakraborty, S.; Zhang, W.; Batson, P. E.; Feldman, L.; Gustafsson, T.; Chhowalla, M. Single Atomic Vacancy Catalysis. *ACS Nano* **2019**, *13*, 9958–9964.

(9) Micevic, A.; Pettinger, N.; Hötger, A.; Sigl, L.; Florian, M.; Taniguchi, T.; Watanabe, K.; Müller, K.; Finley, J. J.; Kastl, C.; Holleitner, A. W. On-Demand Generation of Optically Active Defects in Monolayer WS_2 by a Focused Helium Ion Beam. *Appl. Phys. Lett.* **2022**, *121*, 183101.

(10) Zhang, Z.; Liang, H.; Loh, L.; Chen, Y.; Chen, Y.; Watanabe, K.; Taniguchi, T.; Quek, S. Y.; Bosman, M.; Bettiol, A. A.; Eda, G. Optically Active Chalcogen Vacancies in Monolayer Semiconductors. *Adv. Opt. Mater.* **2022**, *10*, 2201350.

(11) Wang, X.; Pettes, M. T.; Wang, Y.; Zhu, J.-X.; Dhall, R.; Song, C.; Jones, A. C.; Ciston, J.; Yoo, J. Enhanced Exciton-to-Trion Conversion by Proton Irradiation of Atomically Thin WS_2. *Nano Lett.* **2023**, *23*, 3754–3761.

(12) Parto, K.; Azzam, S. I.; Banerjee, K.; Moody, G. Defect and Strain Engineering of Monolayer WSe_2 Enables Site-Controlled Single-Photon Emission up to 150 K. *Nat. Commun.* **2021**, *12*, 3585.
